# A Case of Burkitt’s Lymphoma Mimicking Peritonitis Carcinomatosa

**DOI:** 10.4274/tjh.galenos.2020.2020.0015

**Published:** 2020-08-28

**Authors:** Deram Büyüktaş, Serdar Örnek, Tülay Tecimer, Burhan Ferhanoğlu

**Affiliations:** 1 Koç University Faculty of Medicine, Department of Hematology, İstanbul, Turkey; 2 American Hospital, Department of Hematology, İstanbul, Turkey; 3 Acıbadem University Faculty of Medicine, Department of Pathology, İstanbul, Turkey

**Keywords:** Burkitt’s lymphoma, Peritonitis carcinomatosa, PETCT

## To the Editor,

**Figure 1:**
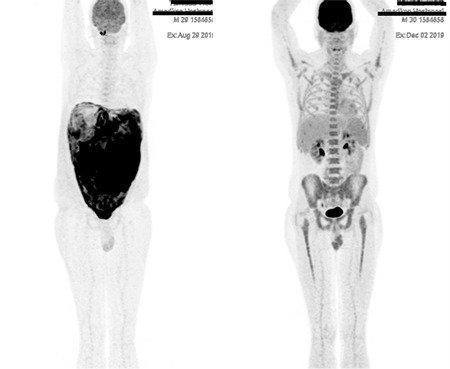
PET-CT before and after treatment. PET-CT: Positron emission tomography-computed tomography

A 30-year-old man was admitted to the hospital with fatigue, fever, nausea, and abdominal distension in August 2019. Laboratory analyses were as follows: white blood cell count, 13,400/µL; absolute neutrophil count, 9,700/µL; absolute lymphocyte count, 2,100/µL; hemoglobin, 14.5 g/dL; platelets, 442,000/µL; C-reactive protein, 15.6 mg/L; lactate dehydrogenase, 186 U/L; ferritin, 927 ng/mL; alanine transaminase, 108 U/L; aspartate transaminase, 245 U/L. Abdominal ultrasound showed massive ascites. Cytospinning of the ascites revealed B-cell non-Hodgkin’s lymphoma. PET-CT showed increased FDG uptake of the whole peritoneum, omentum, and small intestine (Figure 1). Peritonitis carcinomatosa was considered in the differential diagnosis. The patient underwent tru-cut peritoneal biopsy; the findings were consistent with Burkitt’s lymphoma. In immunohistochemical analysis, CD20, CD10, bcl6, and c-myc were positive; CD5, bcl2, CD23, MUM1, and TDT were negative. The Ki-67 index was 99%. FISH analysis for myc/IGH translocation was positive. Bone marrow was normocellular with no sign of lymphoma involvement and conventional cytogenetics showed a normal karyotype: 46, XY [20]. Cerebrospinal fluid cytospinning was also negative for any atypical cells. He was treated with the GMALL protocol [1]. Interim PET was consistent with complete response after four cycles of the regimen (Figure 1). The patient completed the rest of the regimen uneventfully and the final PET-CT did not show any residual disease or recurrence.
